# Mixed anxiety-depressive disorder in Parkinson's disease associated with worse resting state functional response to deep brain stimulation of subthalamic nucleus

**DOI:** 10.1016/j.heliyon.2024.e30698

**Published:** 2024-05-06

**Authors:** Pavel Filip, Andrej Lasica, Tereza Uhrová, Josef Mana, Filip Růžička, Jiří Keller, Karsten Mueller, Kristína Burdová, Dimitra Kiakou, Robert Jech

**Affiliations:** a Department of Neurology, Charles University, First Faculty of Medicine and General University Hospital, Prague, Czech Republic; b Center for Magnetic Resonance Research (CMRR), University of Minnesota, Minneapolis, MN, USA; c Department of Science and Research, Prague College of Psychosocial Studies, Prague, Czech Republic; d Department of Radiology, Na Homolce Hospital, Prague, Czech Republic; e *Third Faculty of Medicine*, *Charles University in Prague*, *Prague*, *Czech Republic*; f Max Planck Institute for Human Cognitive and Brain Sciences, Leipzig, Germany

**Keywords:** Deep brain stimulation, Parkinson's disease, Subthalamic nucleus, Resting-state functional magnetic resonance imaging, Connectivity, Graph theory, Network-based statistics

## Abstract

**Background:**

Parkinson's disease (PD), even though generally perceived as a dominantly motor disorder, is associated with a wide range of non-motor symptoms, including mixed anxiety-depressive disorder (MADD).

**Objectives:**

The aim of the presented study was to determine whether deep brain stimulation (DBS) of the subthalamic nucleus (STN) brings the functional characteristics of non-motor networks closer to the condition detected in healthy population and whether pre-DBS presence of MADD in PD patients was associated with different reaction to this therapeutic modality.

**Methods:**

Resting-state fMRI signature elicited by STN DBS activation and deactivation in 81 PD patients was compared against healthy controls, with the focus on measures of efficiency of information processing and localised subnetwork differences.

**Results:**

While all the MRI metrics showed statistically significant differences between PD patients in DBS OFF condition and healthy controls, none were detected in such a comparison against DBS ON condition. Furthermore, in the post-DBS evaluation, PD patients with MADD in the pre-DBS stage showed no differences in depression scales compared to pre-DBS psychiatrically intact PD patients, but still exhibited lower DBS-related connectivity in a subnetwork encompassing anterior and posterior cingulate, dorsolateral prefrontal and medial temporal cortices.

**Conclusions:**

STN DBS improved all the metrics of interest towards the healthy state, normalising the resting-state MRI signature of PD. Furthermore, pre-DBS presence of MADD, even though clinically silent at post-DBS MRI acquisition, was associated with lower DBS effect in areas highly relevant for depression. This finding points to a possibly latent nature of post-DBS MADD, calling for caution in further follow-up of these patients.

## 1 Introduction

Although Parkinson's disease (PD) is dominantly defined as a motor disorder, it is associated with a wide range of non-motor symptoms, including psychiatric problems. Depression is one of the most common syndromes in PD patients, affecting 30–40 %, with symptoms developing potentially even in the premotor stage of the disease [1]. The diagnosis is based not only on the presence of a persistent negative mood condition, but also on associated disturbances as deficits in attention, psychomotor speed, motivation, sleep and appetite. Furthermore, in most patients, depression coexists with anxiety [2], which - possibly a result of the disease itself or a psychological reaction to the stress associated with PD [3] - substantially contributes to the affection of a broad range of functional domains. Their combination, labelled mixed anxiety-depressive disorder (MADD), is a key determinant of low health-related quality of life in PD patients and increased caregiver burden [4].

Although the exact underlying mechanisms of MADD in PD are not known in full detail, changes in brain structure, signalling neurotransmitters and levels of neurotrophic and inflammatory factors have been suggested as possible contributors [5]. From the neuroanatomic point of view, the most influential model of basal ganglia organisation proposed strictly segregated cortico-subcortico-cortical pathways originating in distinct prefrontal cortical areas [6], and was further developed into a unifying model of combined depression and neurodegeneration in PD [7]. Even though the limits of these excessively schematizing models have since been recognised, there are still several merits to them. They hypothesise about PD-specific factors such as monoaminergic deficits and dysfunctions in frontal subcortical circuits encompassing mainly orbitofrontal cortex, but also anterior temporal cortex and basotemporal limbic circuits, as potential contributors to MADD in PD [5]. Importantly, findings from metabolic, functional, structural imaging studies and autopsies support these previous hypotheses [8,9], and point to complex affections of cortical-limbic networks, dopaminergic prefrontal areas and basal ganglia, but also noradrenergic limbic and brainstem structures.

Considering this wide range of affected structures, it is not surprising that various therapeutic modalities implemented in PD, even though primarily focused on improving motor symptoms, exhibit substantial effects on MADD and other non-motor symptoms as well. Deep brain stimulation (DBS) of the subthalamic nucleus (STN) has been consistently heralded as one of the top therapeutic modalities in advanced PD. Even though its effect on the mood has been subject to scrutinous investigations, given the early reports of increased risk of suicide mostly, but not exclusively, in patients with a history of depression [10], the literature is plagued by conflicting results. In general, STN DBS is reported to have a positive effect on the quality of life and also lead to a moderate improvement of mood aggregate scales [11]. When considered in the context of the ambiguity in the primary driver among all the hypothesised effects of STN DBS on motor improvement [12], a major field of interest meriting further research opens.

To answer the question about the effect of STN DBS on non-motor networks relevant for MADD, the presented study enrolled STN DBS PD patients to undergo resting-state functional MRI (rsfMRI) acquisition with active STN DBS and STN DBS switched off. Several approaches to the rsfMRI analysis were employed (A) to describe the whole non-motor network: graph theory-derived measures – global efficiency and total clustering, but also (B) to search for smaller localised differences and within-network components utilising Network-based statistics (NBS). Moreover, all the included PD patients had undergone an extensive assessment by an experienced psychiatrist specialised in neurodegeneration-related psychiatric disorders before the DBS implantation for the presence of MADD, allowing us to compare whether MADD presents a predictor of further clinical effect of DBS and whether the effect of STN DBS in MADD PD patients exhibited any differences to PD patients without MADD (non-MADD PD) (aim 2). We hypothesised that STN DBS would lead to the normalisation of the network state, binging it closer to the condition detected in healthy controls (HC), and that MADD PD patients would show different rsfMRI patterns when compared to non-MADD PD patients, possibly even defying this normalisation effect.

## 2 Methods

### 2.1 Subjects

In total, 88 PD patients with chronic STN DBS and 20 sex and age-matched HC were enrolled into this study. PD patients met the diagnostic criteria for clinically established PD defined by the Movement Disorders Society [13]. The inclusion criteria for PD patients were bilateral STN DBS implantation at least 3 months before the rsfMRI acquisition and stable programming parameters at least 2 weeks before the rsfMRI acquisition. PD-specific exclusion criteria were the presence of absolute exclusion criteria and red flags for PD as defined by the guidelines [13]. Furthermore, common exclusion criteria for all the subjects were as follows: general contraindications to MRI examination (other than the presence of a full implanted DBS system), substantial vascular or space occupying brain lesions or a neurological and/or psychiatric disorder other than PD and its related complications. The following clinical data were collected for PD patients.

- Before STN DBS implantation (pre-DBS): complex examination by a psychiatrist specialised in organic, neurodegeneration-related psychiatric disorders (T.U.), providing a binarized evaluation of the presence of MADD and Montgomery-Asperg Depression Rating Scale (MADRS) score; examination of cognition (Dementia Rating Scale-2 (DRS-2)); motor examination (either Unified Parkinson's disease Rating Scale (UPDRS), part III (in subjects implanted before the year 2018), or the Movement Disorder Society-sponsored revision of UPDRS (MDS-UPDRS) part III (in subjects implanted after the year) [14].
- At rsfMRI session (post-DBS): disease duration, medication, motor examination (UPDRS or MDS-UPDRS, part III), MADRS, DRS-2, current medication (specifically levodopa equivalent dose and use of antidepressants) and DBS-related parameters (time since the DBS implantation, active contacts, therapy impedance, current/voltage-based stimulation amplitude, pulse width and frequency).

UPDRS scores were converted to MDS-UPDRS values using a previously published approach [15] and a parameter labelled “clinical motor improvement” of DBS was calculated as the ratio of the difference of MDS-UPDRS score, part III before DBS implantation (with antiparkinsonian medication) and post-DBS score (with antiparkinsonian medication and active DBS) to the score before DBS implantation, so that positive values corresponded to clinical improvement. MADRS change was calculated as simple numerical difference between MADRS score at MRI acquisition and pre-DBS MADRS score and labelled “clinical psychiatric improvement”.

The study protocol was approved by the Ethics Committee of the General University Hospital in Prague and every subject signed a written informed consent in accordance with the Declaration of Helsinki.

### 2.2 Imaging protocol and data analysis

For the full imaging and data processing protocol, see the supplementary material.

Briefly, a 1.5 Tesla Siemens Symphony System (Siemens, Erlangen, Germany) was used to acquire a T1-weighted (T1w) structural scan and a rs-fMRI session (gradient-recalled echo echo-planar imaging sequence): in-plane resolution 3 × 3 mm^2^, slice thickness 3 mm, 1 mm interslice gap, 31 slices, TR 3000 ms, TE 51 ms, FA 90°, 200 volumes. PD patients were off antiparkinsonian medication (last l-dopa dose in the evening the day before the MRI acquisition; dopa-agonists and other auxiliary medication as amantadine had been discontinued for at least 3 days) and underwent two rs-fMRI acquisitions, starting randomly either with the DBS system switched on (DBS ON) or switched off (DBS OFF). Each of the two rs-fMRI acquisitions was performed with the participant having been in the relevant DBS state for at least 20 min to allow for the acute effects of DBS to dissipate.

Lead-DBS software [16] was utilised to determine the position of DBS leads and active contacts. The overlap of the volume of tissue activated (VTA) region of interest (ROI) and whole STN, and separately its limbic and associative part was calculated, providing three volumes for each side, subsequently averaged to get the bilateral mean activated volume for each of the above stated ROIs.

rs-fMRI pre-processing included the following steps: slice timing correction, the realignment of the timeseries to correct for subject motion, co-registration to the structural T1w scan combined with T1w-derived warp into the Montreal Neurological Institute (MNI) space and mapping to the standard CIFTI grayordinate space with 2-mm full width at half maximum surface and subcortical volume smoothing, loosely based on the Human Connectome Project (HCP) Minimal Preprocessing Pipeline [17]. The subsequent processing consisted of MELODIC independent component analysis and automatic artefactual components identification via the FIX algorithm, as described in the HCP rs-fMRI pipeline [18].

Processed and masked rs-fMRI data were then parcellated using a combination of HCP-derived cortical parcellation consisting of 180 parcels per hemisphere [19] and resting-state network-based sub-segmentation of Freesurfer-derived subcortical grey matter structures (in total 68 subcortical sub-segments). Only areas most relevant for the hypotheses were considered in further steps, i.e. parcels of insular, medial and lateral temporal cortex, temporo-parieto-occipital junction, inferior parietal cortex, cingulate and prefrontal cortices (excluding somatosensory, motor, auditory and visual cortices). In the subcortical ROIs, thalamus, putamen, caudate, hippocampus and amygdala were selected, but their somatomotor, visual and auditory resting-state network areas were excluded – see Supplementary Fig. 1 for more information. Signal dropouts in the vicinity of the subcutaneous loop of extension cables in the left fronto-parietal area and entry points of implanted leads were masked out utilising a previously described tSNR-based semiquantitative approach [20]. This combination of ROI selection based on their published functions and masking of areas of low data quality yielded 230 rsfMRI signal nodes. FSLNets was used to generate partial correlation matrices regularised using L2-norm Ridge regression. Brain Connectivity Toolbox (BCT) [21] was then utilised to calculate both positive and negative total clustering and global efficiency of this network of interest. And lastly, one-sample *t*-test at predetermined alpha of 0.05 was performed for each inter-node connection of these partial correlation matrices to create sparse matrices for each subject to be fed to the Network-Based Statistics (NBS) toolbox.

### 2.3 Statistical analysis

#### 2.3.1 Demographic and clinical data were summarised with descriptive statistics

The analysis of the Aim 1, the effect of STN DBS on cognition and behaviour-related networks, was based on two repeated-measures general linear models (GLMs): one for the three main BCT parameters (positive and negative total clustering and global efficiency), and one for the sparse connectivity matrices for the NBS analysis as dependent variables. Both GLMs included the session (DBS ON and DBS OFF) as fixed factor, and the following parameters of non-interest: sex, age, time since DBS implantation, clinical motor effect, clinical psychiatric effect, and average combined volume of associative and limbic STN subsection coverage by the VTA. To compare these effects against HC, further four GLMs were constructed: two subject group comparisons (HC vs PD DBS ON, and HC vs PD DBS OFF separately), each with two equivalent GLMs for rs-fMRI parameters of interest (one GLM with three main BCT parameters and one GLM with sparse connectivity matrices for NBS analysis as dependent variables), and with sex and age as covariates of non-interest.

To cover the Aim 2, the comparison between non-MADD and MADD PD patients, two more GLMs were used (one GLM with three main BCT parameters, and one GLM with sparse connectivity matrices for NBS analysis as dependent variables), with PD group (non-MADD and MADD) and rs-fMRI session (DBS ON and DBS OFF) as predictors, and the following parameters of non-interest: sex, age, time since DBS implantation, clinical motor effect, binarized use of antidepressants (yes/no) and average combined volume of associative and limbic STN subsection coverage by VTA. The comparison of clinical parameters of interest between non-MADD and MADD PD patients was based on one further repeated-measures GLM, with MDS-UPDRS and MADRS as dependent variables; group (non-MADD and MADD PD) and session (pre-DBS and post-DBS at the MRI acquisition) as predictors; and the following nuisance covariates: sex, age, time since DBS implantation, binarized use of antidepressants (yes/no) and average combined volume of associative and limbic STN subsection coverage by VTA.

Eventual missing data of covariates of non-interest (see Table 1 for the overview of datapoint availability) were imputed by group mean substitution. The GLMs where parameters with incomplete dataset were entered as dependent variables (comparison of clinical parameters between non-MADD and MADD and correlations of clinical psychiatric improvement) utilised only the limited cohort with full data of interest available (62 PD subjects in total).

**Table 1:** **Basic demographic and clinical information about healthy controls, non-MADD and MADD PD patients.** Data is provided as average [standard deviation]. Parameters calculated only over the available datapoints as presented in the last column labelled “Count” for non-MADD/MADD PD patients, respectively. Abbreviations: DBS – deep brain stimulation; HC – healthy controls; Non-MADD PD – Parkinson's disease without mixed anxiety-depressive disorder; MADD PD – Parkinson's disease with mixed anxiety-depressive disorder at the DBS implantation; F – female; M − male; MADRS – Montgomery-Asberg Depression Rating Scale; STN – subthalamic nucleus.

	HC	Non-MADD PD	MADD PD	non-MADD vs MADD PD	
				P value	Count
Number of subjects	20	39	42		
Age (years)	56.3 [14.6]	59.5 [7.8]	59.7 [7.0]	>0.50	39/42
Sex (count of F/M)	7/13	12/27	13/29	>0.50	39/42
Time since disease onset (years)		12.9 [5.0]	13.4 [4.4]	>0.50	27/24
DBS-related information					
**Time since DBS implantation (months)**		22.4 [28.7]	31.3 [30.0]	0.18	39/42
**Stimulator type** **Infinity/Kinetra/RC Activa]**		18/1/20	15/1/26		39/42
**Stimulation mode [monopolar/bipolar/interleaved]**		38/1/0	40/1/1		39/42
**Constant voltage/constant current mode** **Voltage amplitude (V) (bilat. average)** **Current (mA) (bilat. average)**		10/29 3.3 [0.5] 2.1 [0.8]	16/26 2.7 [0.5] 2.0 [0.6]		39/42
**Pulse width (us)**		64.8 [10.9]	65.9 [11.9]		39/42
**Frequency (Hz)**		129.1 [4.8]	129.3 [12.3]		39/42
**Total electrical energy delivered (μW)**		54.4 [39.1]	51.6 [45.8]	>0.50	35/33
**Impedance (Ω)**		1149.0 [352.1]	1127.3 [260.0]		35/33
Activated volume of STN (mm^3^)		8.7 [11.3]	10.0 [11.0]	>0.50	39/41
Associative subsection (mm^3^)		2.8 [4.4]	2.8 [4.2]	>0.50	39/41
Limbic subsection (mm^3^)		1.4 [2.2]	1.3 [1.6]	>0.50	39/41
Clinical motor improvement (%)		35.0 % [15.3 %]	35.8 % [15.6 %]	>0.50	38/40
MADRS					
**MADRS pre-DBS**		2.9 [2.6]	6.4 [4.0]	**0.01**	38/42
**MADRS post-DBS**		2.3 [3.0]	4.5 [4.2]	0.26	32/31
Time since DBS implantation at post-DBS MADRS (months)		20.7 [17.7]	31.5 [31.3]	0.10	32/31
**Clinical psychiatric improvement**		−0.7 [4.1]	−1.8 [5.8]	0.26	31/31
DRS II					
**DRS II pre-DBS**		139.5 [3.5]	139.2 [2.9]	>0.50	37/42
**DRS II post-DBS**		137.0 [5.9]	137.3 [4.9]	>0.50	37/36
**DRS change**		−2.5 [5.4]	−1.8 [4.6]	0.26	36/36
Medication at MRI session					
**l****-dopa equivalent dose (mg)**		912 [505]	1075 [537]	0.20	39/42
**Antidepressants (% of subjects using any antidepressant)**		33 %	38 %	>0.50	39/42

For the analysis of BCT and clinical parameters, permutation-based non-parametric analysis as implemented in the Permutation Analysis of Linear Models (PALM) package [22] was utilised. Non-parametric combination (NPC) approach across positive/negative total clustering was used to perform joint inference about total clustering (combining positive and negative parameters). For the sparse matrices, NBS toolbox was implemented, with family-wise error (FWE) correction over each network cluster component based on the null distribution of the size of maximal component derived from non-parametric permutation approach. In both PALM and NBS packages, 10,000 permutations were run.

All the results were considered significant at alpha <0.05 with False Discovery Rate (FDR) correction [23] over higher-level comparisons (separately for the aim 1 and aim 2).

## 3 Results

Out of the 88 enrolled PD subjects, five PD subjects were excluded due to substantial atrophy and/or structural changes (e.g. cysts) and two PD subjects were excluded due to DBS hardware problems (broken DBS lead and incorrect DBS lead position). All PD subjects had sufficient brain coverage with good fMRI signal and no subjects exhibited framewise motion beyond 3 mm. Ergo, 81 PD patients with full MRI datasets and 20 HC were considered in the further analyses.

Basic demographic and clinical data, including the availability of individual parameters, is provided in Table 1. No statistically significant differences were found in the age and sex between the three subject groups, and in the time since disease onset, pre-DBS DRS-2, DRS-2 at the time of MRI acquisition, time since DBS implantation and the overlap of VTA and STN between non-MADD and MADD PD subjects (all p values > 0.150, see Table 1 for details). No statistically significant correlation between MADRS and the overlap of VTA and STN ROIs was detected (p > 0.200).

### 3.1 Aim 1 – comparison of DBS on and DBS OFF in PD patients and HC

**Fig. 1:**
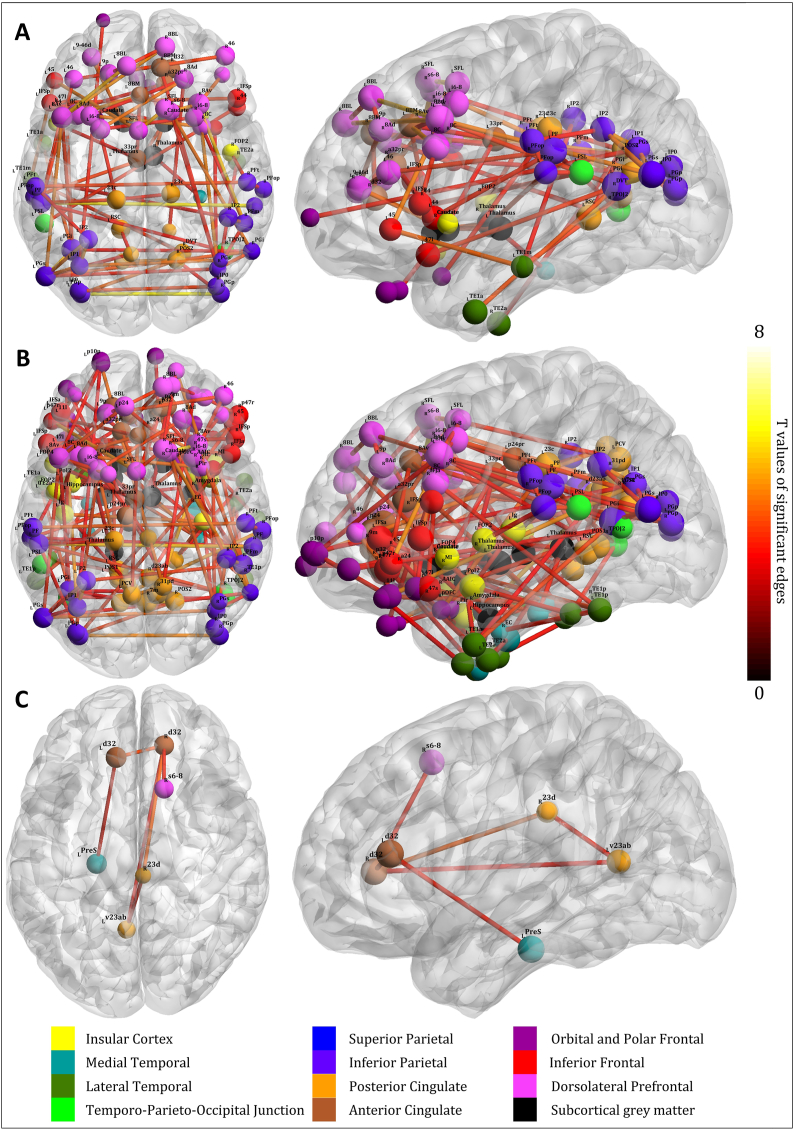
**Network-Based Statistics analysis for Aim 1 and 2.** Edges with higher connectivity in the following contrasts are presented: **A)** healthy controls > Parkinson's disease (PD) DBS OFF. **B)** PD DBS ON > DBS OFF. **C)** Interaction contrast of PD without mixed anxiety-depressive disorder > PD with mixed anxiety-depressive disorder versus DBS ON > DBS OFF. The contrast PD DBS ON vs healthy controls yielded no statistically significant differences. Axial (top) and sagittal (from the left side) views provided. The colour of the edges denotes their statistical significance (T value), see the ribbon at the right side. The colour of the nodes corresponds to a higher cortical area (see the legend below the figure), the size of the node to weighted node strength. Only active nodes relevant for the visualised network presented and in A) and B), only nodes with the sum of T values of their edges >10 are labelled [19]. For the full list of edges, see Supplementary Tables 1 and 2, and Table 3. Abbreviations: L – left; R – right. For the full list of abbreviations and information on individual parcels utilised as nodes, see Ref. [19]. (For interpretation of the references to colour in this figure legend, the reader is referred to the Web version of this article.)

In all the considered metrics (total clustering, global efficiency and NBS analysis), STN DBS brought the rsfMRI signature of the patients closer to the condition seen in HC (see Table 2 for BCT metrics). Generally, while there was a statistically significant difference between PD DBS OFF condition and HC, no similar difference was detected in the comparison between PD DBS ON condition and HC. NBS revealed an extensive network of lower connectivity in PD DBS OFF when compared to HC, consisting of 86 significant edges (see Fig. 1A, Supplementary Table 2 for the full list and Supplementary Table 4 for the T statistic matrix) (p_FDR cor_ < 0.001 for the whole network), encompassing both thalami and caudates, cingulate, temporal, inferior parietal cortices and all the prefrontal cortices.

**Table 2:** **Results for Aim 1 analysis – the comparison between DBS ON and OFF condition in Parkinson's disease patients and between healthy controls – global network parameters (total clustering and global efficiency).** Data is provided as average [standard deviation] for relevant parameters and Fisher's statistic (total clustering)/T statistic (global efficiency) [p value after FDR correction]. For better legibility, both total clustering coefficients and global efficiency values have been multiplied by 10,000. Statistically significant results are marked in bold and with asterisk. Abbreviations: HC – healthy controls; PD – Parkinson's disease; DBS-ON – session with active deep brain stimulation; DBS-OFF – session with inactive deep brain stimulation.

	HC	PD DBS-OFF	PD DBS-ON	HC vs PD DBS OFF	HC vs PD DBS ON	PD DBS OFF vs PD DBS ON
Positive total clustering	98.13 [3.97]	103.44 [8.25]	100.25 [13.26]	30.424 [0.005]*	8.056 [0.304]	17.288 [0.047]*
Negative total clustering	88.06 [5.18]	95.36 [10.85]	91.57 [16.26]			
Global efficiency	475.8 [25.0]	445.3 [60.4]	467.8 [107.4]	2.496 [0.039]*	0.428 [0.695]	1.906 [0.058]

Accordingly, the pairwise comparison between DBS OFF and DBS ON conditions in PD patients detected lower connectivity in an extensive network of 127 edges in the DBS OFF condition (see Fig. 1B. Supplementary Table 3 for the full list and Supplementary Table 5 for the T statistic matrix) (p_FDR cor_ < 0.001 for the whole network), including virtually all the major cortical areas considered in the predetermined mask with the exception of the superior parietal cortex. However, in the BCT analysis, only total clustering comparison yielded significant inter-session difference. Global efficiency failed to survive the post-hoc FDR correction.

### 3.2 Aim 2 – comparison between non-MADD and MADD PD patients

As seen in Table 4, non-MADD PD patients exhibited a 4,0 % and 4.8 % DBS-elicited change of total clustering and global efficiency, respectively, contrary to 2.6 % and 3.1 % change in MADD PD patients. However, when corrected for relevant covariates, the comparison of the response of non-MADD and MADD PD patients to the effect of DBS (interaction of group [non-MADD vs MADD PD] and session [DBS ON vs DBS OFF]) failed to yield statistically significant results (see Table 4). However, NBS analysis revealed a network consisting of anterior and posterior cingulate, dorsolateral prefrontal cortex and medial temporal cortex where non-MADD patients exhibited more significant connectivity increase associated with the DBS ON > OFF contrast than the MADD patients (see Table 3 and Fig. 1C) (p_FDR cor_ = 0.040 for the whole network).

**Table 3:** **Results for Aim 2 analysis – comparison of the response of Parkinson's disease patients with mixed anxiety-depressive disorder and without it to the effect of DBS - Network-Based Statistics analysis.** List of individual edges consisting of a pair of nodes with higher-level anatomical structure and the relevant T value. *P*-value with Family-wise error correction over the subnetwork components and further false discovery rate correction over higher level comparisons. For graphical representation, Abbreviations: L – left; R – right; FWE – Family-Wise Error correction; FDR – False Discovery Rate correction. For more information about the abbreviations denoting individual anatomical subsegments utilised as nodes (here presented in parentheses), see (33).

Node 1	Node 2	T val	p value (FWE + FDR)
Anterior Cingulate (R d32)	Posterior Cingulate (R 23d)	3.919	**0.0398**
**Posterior Cingulate (L v23ab)**	Posterior Cingulate (R 23d)	3.216	
**Dorsolateral Prefrontal Cortex (R s6-8)**	Anterior Cingulate (R d32)	3.141	
**Posterior Cingulate (L v23ab)**	Anterior Cingulate (R d32)	3.063	
**Anterior Cingulate (L d32)**	Anterior Cingulate (R d32)	3.655	
**Medial Temporal (L PreS)**	Anterior Cingulate (L d32)	3.199

**Table 4:** **Results for Aim 2 analysis - comparison of the response of Parkinson's diasease patients with mixed anxiety-depressive disorder and without it to the effect of DBS - full network parameters (total clustering and global efficiency).** Data is provided as average [standard deviation] for relevant parameters and Fisher's statistic (total clustering)/T statistic (global efficiency) [p value after False discovery rate correction]. For better legibility, both total clustering coefficients and global efficiency values have been multiplied by 10,000. No statistically significant results were detected. Abbreviations: HC – healthy controls; MADD - mixed anxiety-depressive disorder; PD – Parkinson's disease; DBS-ON – session with active deep brain stimulation; DBS-OFF – session with inactive deep brain stimulation; FDR - False discovery rate.

	Non-MADD PD			MADD PD			Fisher/	p value (FDR cor.)
	DBS OFF	DBS ON	% Δ	DBS OFF	DBS ON	% Δ	T-stat	
**Positive total clustering**	104.45 [5.88]	100.57 [14.55]	**−4.20 %**	102.49 [9.79]	99.95 [11.75]	**−2.80 %**	4.6833	0.6275
**Negative total clustering**	97.13 [8.15]	92.44 [17.90]	**−3.70 %**	93.72 [12.53]	90.77 [14.32]	**−2.50 %**		
**Global efficiency**	−437.0 [32.3]	−468.5 [115.5]	**−4.80 %**	−453.0 [76.5]	−467.1 [97.9]	**−3.10 %**	−0.7381	0.6275

The analysis of clinical parameters of interest was based only on 31 non-MADD and 31 MADD PD patients due to the incomplete datasets in other subjects. There was no significant difference in the pre-DBS MDS-UPDRS, post-DBS MDS-UPDRS scores or clinical motor improvements among these two groups of patients (p_FDR cor_ > 0.200). As for MADRS, there was a statistically significant difference in the pre-DBS score between non-MADD PD and MADD PD patients (T stat = −3.094, p_FDR cor_ = 0.012). However, no statistically significant difference was detected in the post-DBS MADRS score or clinical psychiatric improvement (p_FDR cor_ > 0.200).

## 4 Discussion

To our knowledge, this is the first large study in PD DBS patients focusing on purely non-motor rsfMRI signature of STN DBS. Furthermore, the enrolled cohort size substantially exceeds the numbers commonly published previously [24], giving a further edge and credibility to the presented outcomes. Our results show that STN DBS brings various rsfMRI characteristics closer to the state detected in healthy controls, further supporting our previous hypothesis on this overarching nature of DBS effect [20]. Moreover, despite the post-DBS lack of difference in MADRS among non-MADD and MADD PD patients, as diagnosed before DBS implantation, rsfMRI analysis revealed that STN DBS leads to substantially lower connectivity in the subnetwork encompassing anterior and posterior cingulate, ventromedial prefrontal cortex and medial prefrontal cortex in MADD PD patients when compared to non-MADD PD patients, pointing to a lingering, clinically silent disparity among these patients. Importantly, these two groups of PD patients showed no statistically significant differences in other parameters of relevance for these findings as cognition, age, DBS-related clinical improvement or medication (see Table 1).

In an attempt for a more precise interpretation of the rsfMRI outcomes, one must especially consider the nature of correlation matrices entered into the analyses – partial correlations after ridge regression were utilised. Simple Pearson's correlation coefficients have been repeatedly shown as unable to provide relevant, self-contained measures of effective connectivity due to their insufficient ability to distinguish between true inter-node connectivity or only relayed signal transmission through one or more internodes [25]. Contrary to that, functional connectivity measures based on partial correlations provide much more viable estimates of direct conditional dependence between brain regions, since the influence of other regions has been regressed out. However, this also brings up a further factor adding to the uncomfortable variability of reported rsfMRI outcomes.

Global efficiency, a factor mathematically expressed as the inverse of the shortest path length, provides an extimation of the information transfer within the network and its efficiency. Our results of lower global efficiency in PD DBS OFF state are well in accord with the previous body of research focused mostly on motor networks in PD [26], even though higher global efficiency in PD has also been reported [27]. This difference probably stems from the analysis approach based on full correlation with subsequent binarization and focus on the full network, contrary to the presented study. What's more important, STN DBS was able to bring this alteration to a level statistically indistinguishable from healthy controls, even when corrected for the motor effect and relative location of the active DBS contact to the STN (see Table 2). Similar outcome may be stated in total clustering coefficient, i.e. the mean clustering coefficient across all the nodes in the network of interest. Since single-node clustering coefficient is defined as the tendency of the node neighbours to connect to each other, the total clustering coefficient is a metric of network segregation, a characteristic necessary for the development of distinct specialised processing patterns [28]. Interestingly, both these parameters detect a substantially higher standard deviation in PD patients – more than double the level of HC in the DBS OFF condition and more than triple the level of HC in the DBS ON condition, which points to non-negligible interindividual variability of STN DBS effect on non-motor networks.

Nonetheless, these parameters at the level of the whole non-motor, non-primary sensory network of interest considered in this study failed to detect small, but possibly clinically relevant differences between non-MADD and MADD PD patients. On the other hand, NBS, a method able to characterize within-network components and uncover smaller, more localised alterations, detected that the DBS-elicited increase in connectivity was of significantly lesser extent in MADD patients than in non-MADD patients in anterior cingulate, posterior cingulate, dorsolateral prefrontal cortex and temporal cortices (see Fig. 1C). All these areas have been listed as potential contributors to major depression in non-neurodegenerative cases [9]. When considered in the context of the absence of statistically significant difference in post-DBS MADRS between non-MADD and MADD PD patients despite their pre-DBS findings (see Table 1), it's possible to speculate on this finding being a latent, clinically silent risk factor for further MADD-related complications. Even more so due to the correction of the analysis for the clinical motor effect, use of medication, precision of STN DBS implantation and time since DBS implantation, which may point to a fixed, underlying failure of coordinated interactions among these crucial areas resistant to both antidepressant medication and effects of STN DBS. Further follow-up studies should be of major interest to evaluate whether MADD diagnosed in the pre-DBS stage truly presents a clinically relevant risk factor for post-DBS complications. We found no differences in the clinical motor effect between non-MADD and MADD patients in our cohort, but it's important to reiterate the complex nature of PD and other neurodegenerative disorders, where motor symptoms are a mere component of the full disease burden. Since not only core symptoms as depressed mood and anhedonia, but also somatic symptoms such as sleep disturbances, loss of appetite, “brain fog” and altered facial expressions are a part of the depression syndrome, the overlap with “standard” PD signs is self-evident.

Several limitations must be taken into account in this study. The inherently altered rsfMRI data quality due to the presence of DBS hardware-related artifacts is a factor difficult to correct for in full extent. However, the main areas of artifacts as the DBS lead entry points and the cortex in their vicinity were masked out in all the subjects due to the deliberate ROI choice focused on non-motor networks and the area under the extension wire loop in the left parietal region was excluded in all the subjects as well. Furthermore, the comparison to HC was intended only to show whether the direction of the eventual DBS ON vs DBS OFF differences conformed with our previous hypothesis of “normalising” effect of DBS or the alteration was more complex. Advanced, zero echo time sequence may be of further interest in the future to avoid the issue of off-resonance artifacts altogether [29]. Secondly, the short time between the DBS ON and OFF sessions, although sufficient to elicit clinical motor effect to disappear or develop, may not allow for the full functional reorganisation of complex areas considered in this study, let alone the neural plasticity-related alterations elicited by DBS. Longitudinal studies starting immediately after the implantation and spanning over the period of several years will be more suitable to disentangle the question of long-term DBS effects on brain function and microstructure. And lastly, MADRS, although commonly used in PD to evaluate the presence of depression, may not be capable of fully appreciating the subtle differences or essential factors characteristic for neurodegeneration-related organic MADD. This may partly explain the absence of any correlation of MADRS and the STN – VTA overlap despite the previous reports of hypomanic effects of the stimulation of ventral STN parts [30]. Long years of experience and training are required for a psychiatrist to reach sufficient confidence and appreciate peculiarities of these patients highly relevant for the management of their psychiatric problems.

## 5 Conclusion

STN DBS leads to the restoration of several rsfMRI metrics towards the state seen in HC not only in the motor networks, but also in areas highly relevant for cognition and mood. Furthermore, while PD patients diagnosed with MADD before DBS implantation failed to show statistically significant difference in MADRS score against non-MADD PD patients post-DBS, their reaction to this restorative effect of STN DBS in multiple cortical areas highly relevant for depression was significantly lower than that of non-MADD PD patients, pointing to potential clinically silent difference and eventual risk factor in these patients. All in all, MADD PD patients require long-term psychiatric support and follow-up, with individualised multimodal approach and caution against eventual flare-ups of clinically latent non-motor symptoms.

## Funding statement

Support was provided by the Czech 10.13039/100009647Ministry of Health (AZV NV19-04-00233) and the 10.13039/501100016366General University Hospital in Prague (MH CZ-DRO-VFN64165) and Na Homolce Hospital (MH CZ-DRO-NNH 23884). In addition, this project has received funding from the European Union's 10.13039/501100007601Horizon 2020 research and innovation programme under the JPND2020-568-028 – Neuripides, National Institute for Neurological Research, Czech Republic, Programme EXCELES (ID project No. LX22NPO5107) and Charles University, Czech Republic – Cooperatio Program in Neuroscience.

## Data availability

The MRI datasets of the presented study are not publicly available due to the sensitive nature and data privacy regulations related to patient data. However, they are available from the corresponding author upon reasonable request.

**Code availability:** not applicable.

**Ethics approval:** The study protocol was approved by the ethics committee of the General University Hospital in Prague, Czech Republic.

**Consent to participate:** Each subject provided a written informed consent in accordance with the Declaration of Helsinki.

## CRediT authorship contribution statement

**Pavel Filip:** Writing – original draft, Software, Methodology, Formal analysis. **Andrej Lasica:** Writing – review & editing, Data curation. **Tereza Uhrová:** Writing – review & editing, Conceptualization. **Josef Mana:** Writing – review & editing, Methodology, Investigation, Conceptualization. **Filip Růžička:** Writing – review & editing. **Jiří Keller:** Writing – review & editing, Data curation. **Karsten Mueller:** Writing – review & editing. **Kristína Burdová:** Writing – review & editing, Data curation. **Dimitra Kiakou:** Writing – review & editing, Data curation. **Robert Jech:** Writing – review & editing, Funding acquisition, Conceptualization.

## Declaration of competing interest

There are no potential conflicts of interests and no financial relationships regarding this paper which could bias this work.
