# Supplementary material for: Mixed anxiety-depressive disorder in Parkinson's disease associated with worse resting state functional response to deep brain stimulation of subthalamic nucleus
Source: Heliyon. 2024 May 6;10(10):e30698. doi: 10.1016/j.heliyon.2024.e30698 (PMC11109721; doi:10.1016/j.heliyon.2024.e30698)

**Supplementary figure 1:** Masking based on the choice of non-motor, non-primary sensory regions of interest (yellow colour, inclusive mask) and on temporal signal-to-noise ratio (red colour, exclusive mask) to mitigate the effect of signal dropouts and artifacts related to DBS hardware. Cortical areas presented as CIFTI greyordinate surface maps (left side of the picture). Subcortical structures shown in 4 slices z = 20, 7, -6, -19 in MNI coordinate system (right side of the picture). Combined parcellation based on HCP-derived cortical parcellation consisting of 180 parcels per hemisphere and resting-state network-based sub-segmentation of Freesurfer-derived subcortical grey matter structures (in total 68 subcortical sub-segments) – see Methods for more information.


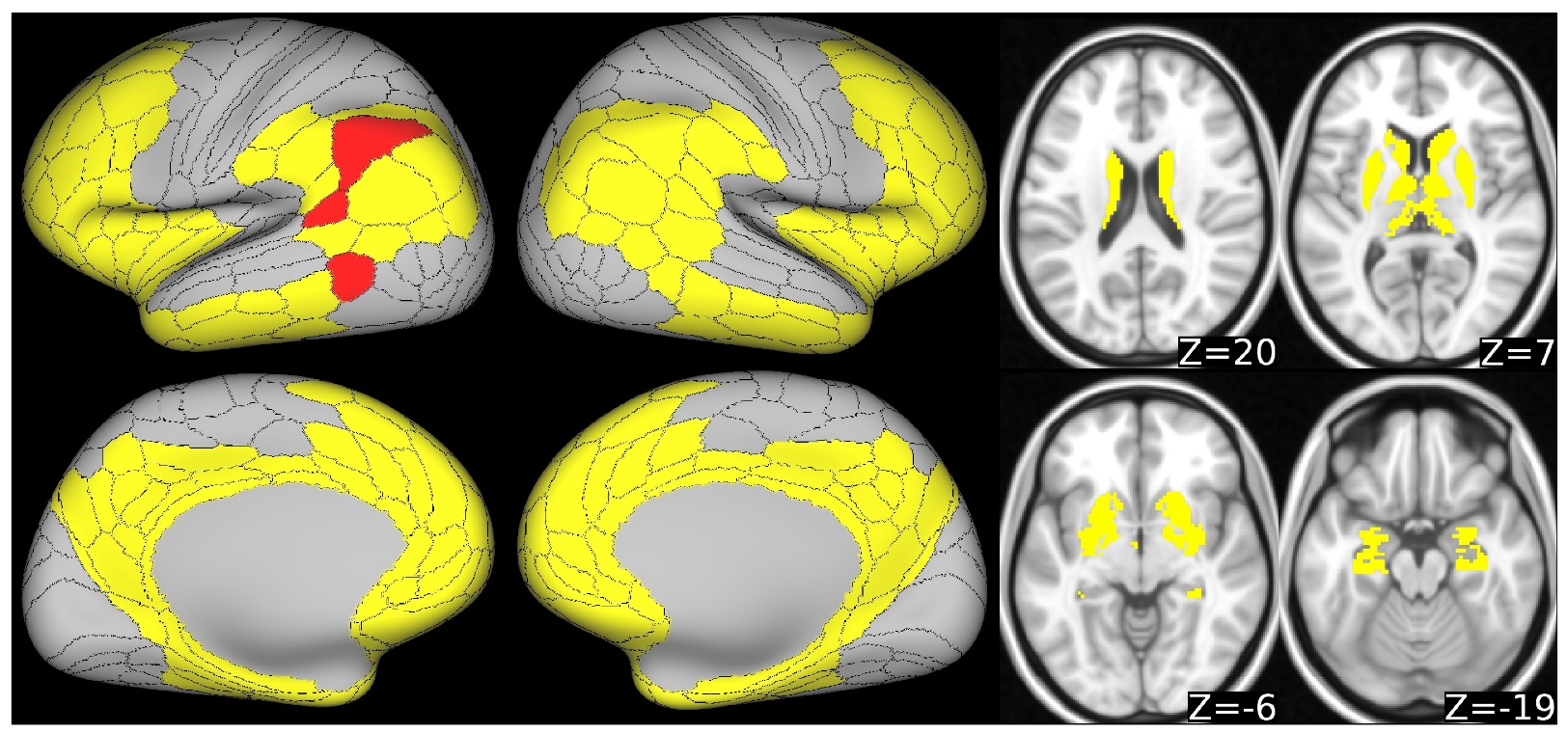

Supplement: Multimedia component 1 [file mmc1.docx]
