# Supplementary material for: Mixed anxiety-depressive disorder in Parkinson's disease associated with worse resting state functional response to deep brain stimulation of subthalamic nucleus
Source: Heliyon. 2024 May 6;10(10):e30698. doi: 10.1016/j.heliyon.2024.e30698 (PMC11109721; doi:10.1016/j.heliyon.2024.e30698)
Supplement: Multimedia component 2 [file mmc2.docx]

## Imaging protocol

MRI acquisition was performed using a 1.5T MAGNETOM Avanto scanner (Siemens, Erlangen, Germany). The protocol included a T1-weighted (T1w) scan with magnetisation-prepared rapid gradient echoes (MPRAGE) sequence with 1.0 mm isotropic resolution, repetition time (TR) of 2,140 ms, inversion time (TI) of 1,100 ms, echo time (TE) of 3.93 ms and flip angle (FA) of 15°. T2*-weighted scans sensitive to the blood oxygenation level dependent contrast were utilised for the rs-fMRI acquisition with the following protocol: gradient-recalled echo (GRE) echo-planar imaging (EPI) sequence, TR 3,000 ms, TE 51 ms, FA 90°, antero-posterior phase-encoding direction; in-plane resolution 3 × 3 mm, slice thickness 3 mm, inter-slice gap 1 mm, with total of 31 axial slices covering the whole brain, 200 rs-fMRI volumes were acquired over the period of 10 minutes. Throughout the rs-fMRI session, each participant was asked to lie motionlessly with the eyes open and fixating on a cross positioned in the middle of the visual field. Furthermore, wakefulness of each subject was continuously monitored with an MRI compatible 12M camera (MRC Systems, Heidelberg, Germany).

HC underwent this protocol in the above-described manner. PD subjects underwent MRI acquisition without antiparkinsonian medication. Eventual dopa-agonist medication had been discontinued at least 3 days before the MRI acquisition and the last L-dopa dose was administered in the evening of the day preceding the MRI session. No other medication alterations were introduced as part of the MRI protocol. Furthermore, each PD subject underwent two rs-fMRI acquisitions, starting randomly either with the DBS system switched on (DBS ON) or switched off (DBS OFF). Each of the two rs-fMRI acquisitions was performed with the participant having been in the relevant DBS state for at least 20 minutes (i.e. the two rs-fMRI sessions were separated by at least 20 minutes).

## DBS electrode position analysis

This processing step used the Lead-DBS software [1] (version 2.5.3) with the enhanced workflow [2]. T1w scans from the above described research MRI protocol were co-registered to the pre-operative T1w scans (see Supplementary table 1 for relevant information on preoperative T1w protocols) with the refinement based on the “brain shift correction” module [3]. Afterwards, the preoperative T1w scan was utilised to generate a warp matrix to the Montreal Neurological Institute (MNI) space with diffeomorphic registration algorithm from Advanced Normalization Tools and subcortical refinement [2]. This matrix was then used to transform the post-DBS research T1w scan into the MNI space as well. Automatic and manual pre-localisation with subsequent manual refinement of electrode trajectories was preformed and electric fields around the active contacts based on the clinical DBS settings (as determined by the attending neurologist) were estimated using the finite element approach and magnitude thresholding of the electric field gradient at the level of 0.2 V/mm [4]. In the last step, the overlap of the volume of tissue activated (VTA) region of interest (ROI) and whole STN, and separately its limbic and associative part was calculated, providing three volumes for each side. These bilateral values were averaged to get the mean activated volume for each of the above stated ROIs.

## Structural MRI data analysis

The structural image processing pipeline was based on the research T1w scans, i.e. the pre-operative T1w scans of PD subjects were not utilised here. In PD subjects, the first step was “lesion-filling” [5] to cover DBS lead artifacts with intensities close to artifact neighbourhood and thus enable the susbsequent processing. This approach utilised manually created masks of DBS lead trajectories and FAST-derived (FMRIB Automated Segmentation Tool as implemented in FMRIB Software Library (FSL), version 6.0 [6] ) white matter mask. The following steps are shared by HC and PD processing pipeline: intensity normalisation over white and grey matter [7], alignment to MNI space (1-mm isotropic voxel template) with rigid-body transformation and a rough initial brain extraction based on non-linear (FSL FNIRT) registration of the T1w image to the MNI template. The lesion-masked normalised T1w image, brain mask and manually selected control points around the DBS lead insertion into the brain (PD subjects only) were fed into the CUDA (Computer Unified Device Architecture)-enabled version of FreeSurfer 6.0. The brain mask generated by FreeSurfer segmentation was then utilised for the final MNI space non-linear warp and the native-mesh surfaces of each subject were registered to the Conte69 population-average surfaces and the 2-mm standard Connectivity Informatics Technology Initiative (CIFTI) grayordinate space for the following fMRI pipeline [8].

## rs-fMRI analysis

rs-fMRI data pre-processing pipeline was loosely based on the Human Connectome Project (HCP) Minimal Preprocessing Pipeline [8]. It included slice timing correction, 3D rigid-body realignment of each frame of the timeseries to the first scan (FSL 6.0 MCFLIRT) to correct for subject motion and co-registeration to the structural T1w scan using mri_robust_register-initialized BB-register algorithm [9]. The above stated two transforms were then combined with the MNI warp matrix derived in the structural data processing to transform every frame from the original timeseries to 2-mm MNI space with a single spline interpolation. Afterwards, the volume timeseries was mapped to the standard CIFTI grayordinate space with the partial volume-weighted ribbon-constrained volume to surface mapping algorithm, excluding voxels with locally high coefficient of variation in the timeseries, and regularised with 2-mm full width at half maximum surface and subcortical volume smoothing. The next steps were based on the HCP rs-fMRI pipeline [10] and included independent component analysis (FSL MELODIC) and automatic artefactual components identification (FIX algorithm) [11]. General FIX package training data was used for the automatic classification and the output was manually checked and corrected by an experienced operator (P.F.), with subsequent “non-aggressive” regression of FIX-defined artefactual MELODIC components and motion-related time-courses.

Afterwards, the outputs were visually evaluated by an experienced operator (P.F.) for the adequacy of FreeSurfer surface reconstruction and segmentation (inaccuracies were corrected manually in 2 HC and 16 PD subjects), co-registration, motion correction and signal dropouts. Quantitative quality control parameters included Foreground-Background Energy Ratio, temporal signal-to-noise ratio (tSNR) over the whole brain of raw data and from fully processed data, brain coverage ratio by voxels with tSNR higher than 10 in MNI space and framewise voxel displacement. Out of the 88 enrolled PD subjects, five PD subjects were excluded due to substantial atrophy and/or structural changes (e.g. cysts) and two PD subjects were excluded due to DBS hardware problems (broken DBS lead and incorrect DBS lead position). All PD subjects had sufficient brain coverage with good fMRI signal and no subjects exhibited framewise motion beyond 3 mm. Ergo, 81 PD patients with full MRI datasets and 20 HC were considered in the further analyses.

Furthermore, signal dropouts in the vicinity of the subcutaneous loop of extension cables in the left fronto-parietal area and entry points of implanted leads were masked out utilising a previously described, tSNR-based semiquantitative approach [12]. The acquired binary mask was then utilised for all the acquisitions, including HC data, to allow for inter-group comparisons of parameters of interest.

Processed and masked rs-fMRI data were then parcellated using a combination of HCP cortical parcellation (180 parcels per hemisphere) [13] and resting-state network-based sub-segmentation of subcortical grey matter structures as extracted by FreeSurfer. Cole-Anticevic Brain Network Parcellation [14] of 12 resting-state networks was utilised in this step and the output subsegments were size-thresholded at the level of 50 voxels, yielding 68 subcortical ROIs. Furthermore, only areas most relevant for the hypotheses were considered in further steps, i.e. parcels of insular, medial and lateral temporal cortex, temporo-parieto-occipital junction, inferior parietal cortex, cingulate and prefrontal cortices (excluding somatosensory, motor, auditory and visual cortices). In the subcortical ROIs, thalamus, putamen, caudate, hippocampus and amygdala were selected, but their somatomotor, visual and auditory resting-state network areas were excluded – see Figure 1 for more information. This combination of ROI selection based on their published functions and masking of areas of low data quality yielded 230 rsfMRI signal nodes. FSLNets was used to generate partial correlation matrices regularised using L2-norm Ridge regression. Brain Connectivity Toolbox (BCT) [15] was then utilised to calculate both positive and negative total clustering and global efficiency of this network of interest. And lastly, one-sample t-test at predetermined alpha of 0.05 was performed for each inter-node connection of these partial correlation matrices over the all the subjects to exclude edges which did not differ significantly from zero. The aim was to create sparse matrices for each subject to be fed to the Network-Based Statistics (NBS) toolbox to provide more localised information on the nature of eventual alterations.

## References

[1] Horn A, Kühn AA. Lead-DBS: a toolbox for deep brain stimulation electrode localizations and visualizations. Neuroimage 2015;107:127–35.

[2] Horn A, Li N, Dembek TA, Kappel A, Boulay C, Ewert S, et al. Lead-DBS v2: Towards a comprehensive pipeline for deep brain stimulation imaging. Neuroimage 2019;184:293–316.

[3] Schönecker T, Kupsch A, Kühn AA, Schneider G-H, Hoffmann K-T. Automated optimization of subcortical cerebral MR imaging- atlas coregistration for improved postoperative electrode localization in deep brain stimulation. Am J Neuroradiol 2009;30:1914–21.

[4] Vasques X, Cif L, Hess O, Gavarini S, Mennessier G, Coubes P. Stereotactic model of the electrical distribution within the internal globus pallidus during deep brain stimulation. J Comput Neurosci 2009;26:109.

[5] Battaglini M, Jenkinson M, De Stefano N. Evaluating and reducing the impact of white matter lesions on brain volume measurements. Hum Brain Mapp 2012;33:2062–71.

[6] Jenkinson M, Beckmann CF, Behrens TE, Woolrich MW, Smith SM. Fsl. Neuroimage 2012;62:782–90.

[7] Cox RW. AFNI: software for analysis and visualization of functional magnetic resonance neuroimages. Comput Biomed Res 1996;29:162–73.

[8] Glasser MF, Sotiropoulos SN, Wilson JA, Coalson TS, Fischl B, Andersson JL, et al. The minimal preprocessing pipelines for the Human Connectome Project. Neuroimage 2013;80:105–24.

[9] Greve DN, Fischl B. Accurate and Robust Brain Image Alignment using Boundary-based Registration. NeuroImage 2009;48:63–72. https://doi.org/10.1016/j.neuroimage.2009.06.060.

[10] Smith SM, Beckmann CF, Andersson J, Auerbach EJ, Bijsterbosch J, Douaud G, et al. Resting-state fMRI in the human connectome project. Neuroimage 2013;80:144–68.

[11] Salimi-Khorshidi G, Douaud G, Beckmann CF, Glasser MF, Griffanti L, Smith SM. Automatic denoising of functional MRI data: combining independent component analysis and hierarchical fusion of classifiers. Neuroimage 2014;90:449–68.

[12] Filip P, Jech R, Fečíková A, Havránková P, R\uužička F, Mueller K, et al. Restoration of functional network state towards more physiological condition as the correlate of clinical effects of pallidal deep brain stimulation in dystonia. Brain Stimulat 2022;15:1269–78.

[13] Glasser MF, Coalson TS, Robinson EC, Hacker CD, Harwell J, Yacoub E, et al. A multi-modal parcellation of human cerebral cortex. Nature 2016;536:171–8.

[14] Ji JL, Spronk M, Kulkarni K, Repovš G, Anticevic A, Cole MW. Mapping the human brain’s cortical-subcortical functional network organization. NeuroImage 2019;185:35–57. https://doi.org/10.1016/j.neuroimage.2018.10.006.

[15] Rubinov M, Sporns O. Complex network measures of brain connectivity: Uses and interpretations. NeuroImage 2010;52:1059–69. https://doi.org/10.1016/j.neuroimage.2009.10.003.
